# Inflammogenic effect of well-characterized fullerenes in inhalation and intratracheal instillation studies

**DOI:** 10.1186/1743-8977-7-4

**Published:** 2010-03-14

**Authors:** Yasuo Morimoto, Masami Hirohashi, Akira Ogami, Takako Oyabu, Toshihiko Myojo, Ken-ichiro Nishi, Chikara Kadoya, Motoi Todoroki, Makoto Yamamoto, Masahiro Murakami, Manabu Shimada, Wei-Ning Wang, Kazuhiro Yamamoto, Katsuhide Fujita, Shigehisa Endoh, Kunio Uchida, Naohide Shinohara, Junko Nakanishi, Isamu Tanaka

**Affiliations:** 1 Institute of Industrial Ecological Sciences, University of Occupational and Environmental Health, Japan. Kitakyushu, Japan; 2 Hiroshima University. Hiroshima, Japan; 3 National Institute of Advanced Industrial Science and Technology, Tsukuba, Japan

## Abstract

**Background:**

We used fullerenes, whose dispersion at the nano-level was stabilized by grinding in nitrogen gas in an agitation mill, to conduct an intratracheal instillation study and an inhalation exposure study. Fullerenes were individually dispersed in distilled water including 0.1% Tween 80, and the diameter of the fullerenes was 33 nm. These suspensions were directly injected as a solution in the intratracheal instillation study. The reference material was nickel oxide in distilled water. Wistar male rats intratracheally received a dose of 0.1 mg, 0.2 mg, or 1 mg of fullerenes and were sacrificed after 3 days, 1 week, 1 month, 3 months, and 6 months. In the inhalation study, Wistar rats were exposed to fullerene agglomerates (diameter: 96 ± 5 nm; 0.12 ± 0.03 mg/m^3^; 6 hours/days for 5 days/week) for 4 weeks and were sacrificed at 3 days, 1 month, and 3 months after the end of exposure. The inflammatory responses and gene expression of cytokine-induced neutrophil chemoattractants (CINCs) were examined in rat lungs in both studies.

**Results:**

In the intratracheal instillation study, both the 0.1 mg and 0.2 mg fullerene groups did not show a significant increase of the total cell and neutrophil count in BALF or in the expression of CINC-1,-2αβ and-3 in the lung, while the high-dose, 1 mg group only showed a transient significant increase of neutrophils and expression of CINC-1,-2αβ and -3. In the inhalation study, there were no increases of total cell and neutrophil count in BALF, CINC-1,-2αβ and-3 in the fullerene group.

**Conclusion:**

These data in intratracheal instillation and inhalation studies suggested that well-dispersed fullerenes do not have strong potential of neutrophil inflammation.

## Background

Nanomaterials are typically defined as structure components with at least one dimension less than 100 nm, and possess superior characteristics that conventional materials do not have, thus they are internationally and aggressively researched and developed. Of the nanomaterials, fullerene is a generic term for clusters consisting of many carbon atoms and a typical fullerene is C60 that has a soccer-ball structure with 60 carbon atoms [1]. Fullerenes have a multitude of applications, including energy device material, super conducting devices, magnets, and catalysts, and they can be mass-produced by a combustion method. Thus the demand for fullerenes is expected to rise. It is unknown whether fullerenes cause lung injury or not and if so, what its mechanism is. One of the causes of this inconsistent study result of industrial nanomaterials is the strong agglomerability of nanoparticles [2,3]. Nanoparticles agglomerate by Van Der Waals forces and electrostatic effect soon after production in gaseous and liquid phases to become larger agglomerates. It is expected that the formation of larger agglomerates may lead to the formation of bronchial and pulmonary artificial granuloma in animal exposure studies [2,3], and thereby death from suffocation, and also may lead to the quantitative and qualitative differences of biological response of its own. There is a report that dispersed nanomaterials were more toxic for bacteria than nanomaterial aggregates [4]. Moreover, unless an experiment examines the dispersed nanoparticles, any experiment in which animals are exposed to undispersed nanoparticles cannot eliminate the possibility that the result is caused by the influence of agglomerates, even if dispersed particles are observed *in vivo*. For this reason, the characterization of nanoparticles is needed; in particular, it is necessary to confirm that the agglomerates are at the nano-size level under the exposure conditions of animal experiments, i.e. inside the inhalation chamber for inhalation studies and in the instillation liquid for intratracheal instillation studies. We controlled the size of fullerene particles at the nano-level by grinding them in nitrogen gas in an agitation mill to obtain a stable dispersion and realize the animal fullerene exposure study at the nano-size level [5].

Pulmonary injury from dust is supposedly caused by inflammation through free-radicals produced by the phagocytosis of dust and fibrosis by abnormal repair [6-8]. In the process of such pulmonary injury, it is suggested that a persistent or progressive inflammation mainly by neutrophils causes irreversible chronic lesions such as fibrosis and tumors. This is an important part of the process of pulmonary injury due to asbestos, silica, and metal nanoparticles [9-12]. The important cytokines involved in inflammation by neutrophils are chemokines such as cytokine-induced neutrophil chemoattractant (CINC) [13,14]. CINC, which belongs to the CXC chemokine family and consists of CINC-1, CINC-2αβ, and CINC-3, not only stimulates the neutrophil chemotactic activity but also enhances phagocytosis, induces adhesion molecules, and activates the function of neutrophils. Additionally, studies have reported an increased expression of CINC in a pulmonary injury animal model by diesel particles and metal nanoparticles [15,16], suggesting that fine particles including nanoparticles are closely related to pulmonary inflammation through CINCs. Accordingly, in order to study pulmonary injury caused by fullerenes, we developed an intratracheal instillation and inhalation animal exposure model to examine neutrophil infiltration and CINC expression in the lung.

## Results

### 1) Characterization of fullerenes (Figure 1)

**Figure 1:**
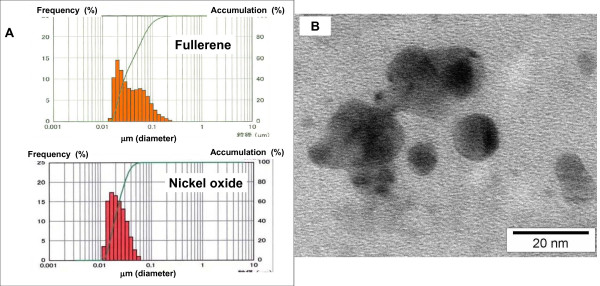
**A: Particle size distribution determined by dynamic light scattering**. Average diameter in the suspension of fullerenes is 33 nm. B; Transmission electron microscope image of fullerene nanoparticles in suspension.

The mean diameter based on the volume and mass of fullerenes in distilled water including 0.1% Tween 80 and nickel oxide in distilled water without any detergents was 33 nm, and 26 nm respectively. The diameter of fullerene nanoparticles by transmission electron microscope (TEM) was almost same level. These suspensions were directly injected as a solution in the intratracheal instillation study, and were also used for generation of fullerene nanoparticles in the inhalation study.

### 2) Intratracheal instillation study

#### Cell count and alkaline phosphatase (ALP) in broncho-alveolar lavage fluid (BALF) (Figure 2)

**Figure 2:**
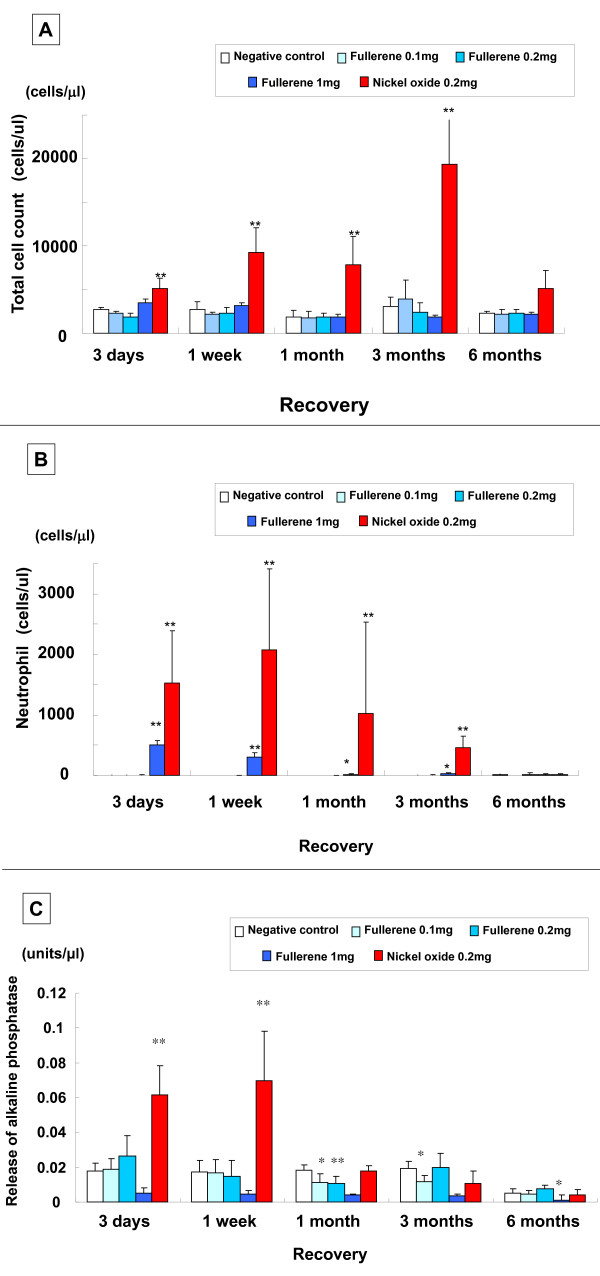
**Analysis of BALF in the intratracheal instillation study**. (A) Total cell count. (B) Neutrophil cell count. (C) Alkaline phosphatase. Each column and bar represents the mean ± standard deviation of five rats. An asterisk indicates a statistically significant difference of P < 0.05 compared to each negative control group; double asterisk, of P < 0.01 compared to each negative control group. A high dose of fullerenes induced transient inflammatory response, and a low dose of fullerenes did not.

In comparison with the negative control group (distilled water including 0.1% Tween 80), the total cell count and neutrophil count did not significantly increase in the fullerene 0.1 mg and 0.2 mg (low dose) groups and the 1 mg (high dose) group. The neutrophil count significantly increased over a period of 3 days to 3 months in the high dose group; however, the increase was slight after 1 month and 3 months. On the other hand, the 0.2 mg nickel oxide group showed a significant increase in the total cell count and neutrophil count over a period of 3 days to 3 months. A significant ALP release was observed from 3 days to 1 week in the nickel oxide group, while no significant release was observed in the fullerene groups.

#### CINC concentration in the lung (Figure 3)

**Figure 3:**
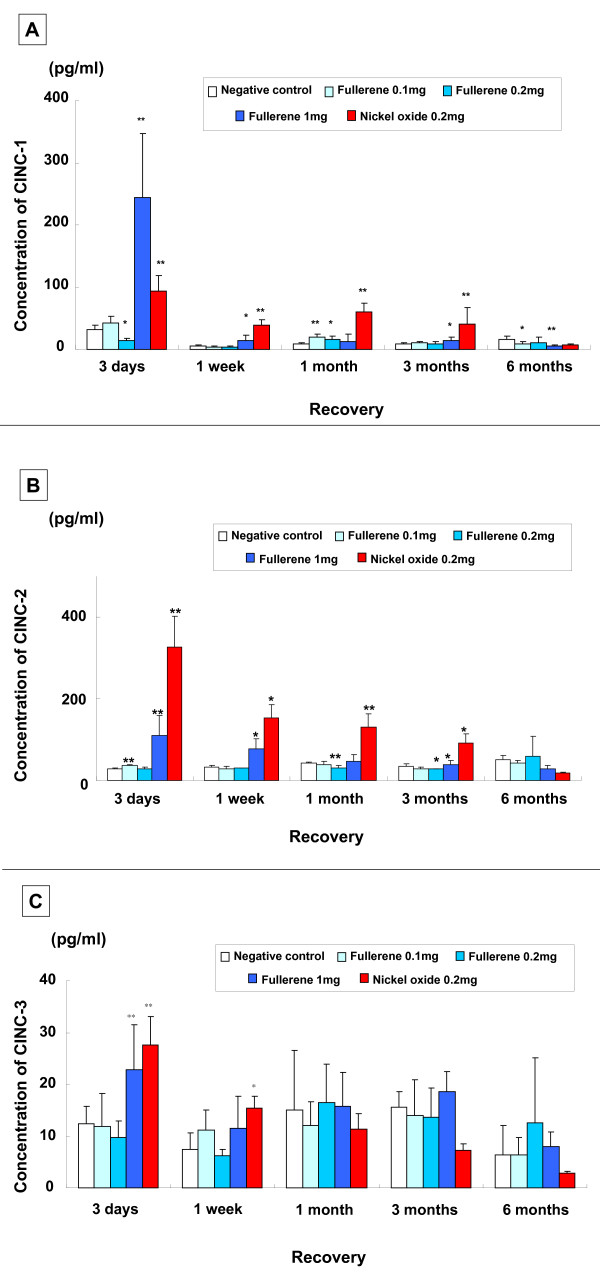
**Concentration of CINC in rat lungs in the intratracheal instillation study**. (A) CINC-1. (B) CINC-2αβ. (C) CINC-3. Each column and bar represents the mean ± standard deviation of five rats. An asterisk indicates a statistically significant difference of P < 0.05 compared to each negative control group; double asterisk, of P < 0.01 comparted to each negative control group. A low dose of fullerenes did not induce concentration of CINC-1 and CINC-2αβ, and a high dose of fullerenes did induce transient increase.

A transient and significant, though mild, increase in CINC-1 concentration was found after 1 month in the 0.1 mg and 0.2 mg fullerene groups. The 1 mg instillation group revealed a significant increase after 3 days; the significance of this increase was lower than that of the nickel oxide group; however, the concentration was high and decreased thereafter. There was a mild increase after 3 months and no increase after 6 months.

As for CINC-2αβ concentration, there was a persistent and significant increase from 3 days to 3 months in the 0.2 mg nickel oxide group. The 1 mg fullerene group showed a significant increase after 3 days; however the concentration was high and decreased thereafter to nearly the same level as the negative control groups after 1 month. As for CINC-3 concentration, there was a significant increase at 3 days and 1 week in the nickel oxide group and at 3 days in the 1 mg fullerene group, while no significant increase was observed in other periods or in other groups.

#### Gene expression of CINC mRNA in the lung (Figure 4)

**Figure 4:**
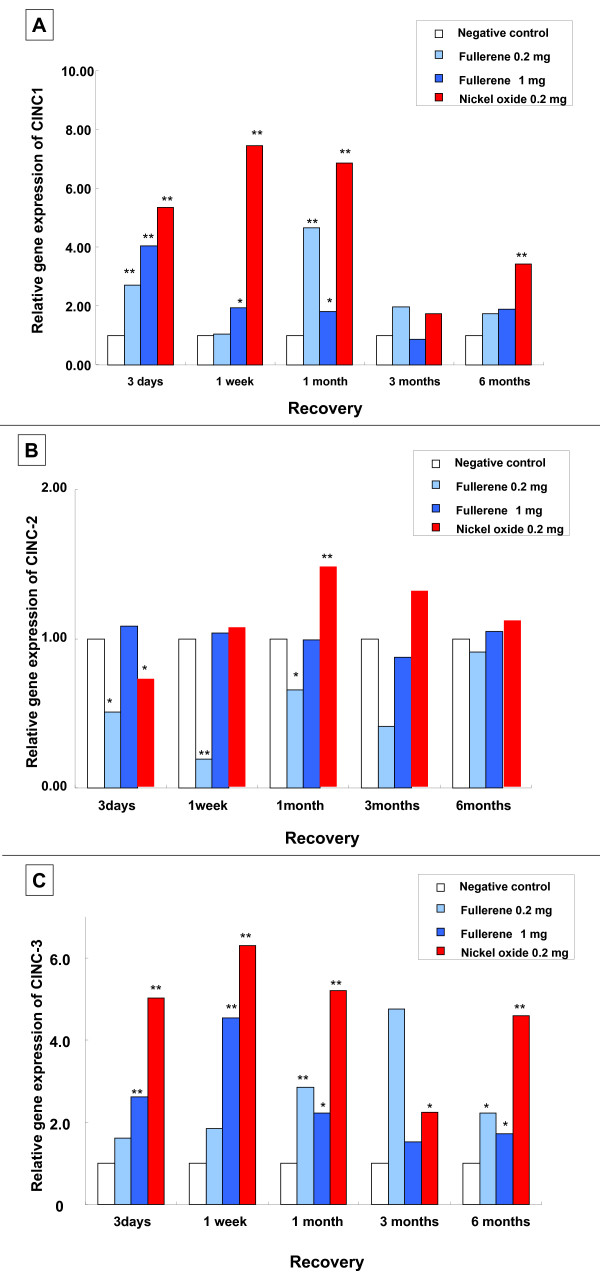
**Gene expression of CINC mRNA in rat lungs in the intratracheal instillation study**. (A) CINC-1. (B) CINC-2αβ. (C) CINC-3. Relative gene expression means 2^-ΔΔCT^. (ΔΔC_T_: the difference of the average ΔC_T _value of the fullerene-exposed group and the average ΔC_T _of the negative control group). An asterisk indicates a statistically significant difference of P < 0.05 compared to each negative control group; double asterisk, of P < 0.01 compared to each negative control group. Fullerene nanoparticles transiently induced the gene expression of CINC-1 and CINC-2αβ in the lung. Nickel oxide nanoparticles (reference material) persistently increased in the gene expression of CINC-1 and CINC-2αβ mRNA not only in the acute phase but chronic phase.

An increased gene expression in CINC-1 in fullerene groups was observed from 3 days to 1 month in the 0.2 mg and the 1 mg instillation groups. CINC-2αβ gene expression tended to increase after 1 month in the 0.2 mg nickel oxide group. No consistent change of CINC-2αβ gene expression was observed in the 0.2 mg and 1 mg fullerene groups. An increased expression in CINC-3 was also observed in the 0.2 mg and 1 mg fullerene groups, however the increase tended to be lower than that of the nickel oxide group.

#### Histopathological changes in the lungs (Figure 5, 6)

**Figure 5:**
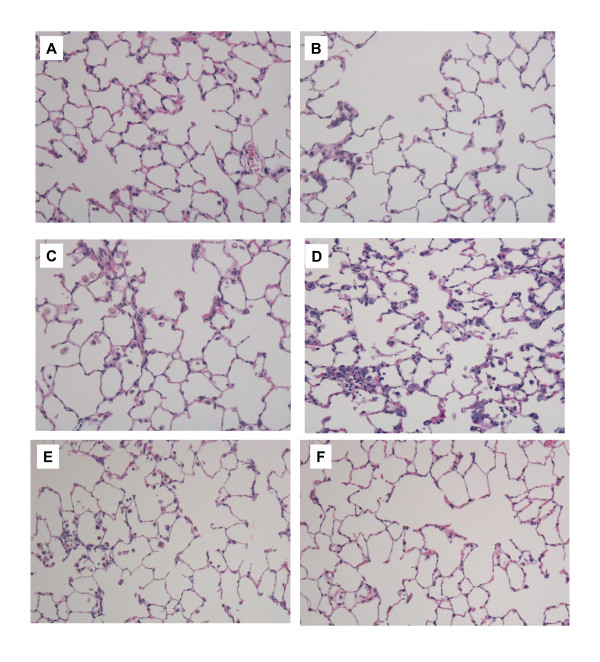
**Hematoxylin and eosin staining of lung sections after intratracheal instillation of fullerenes**. Magnification ×100. (A) Negative control at 3 days (B) 0.2 mg fullerene-exposed lung at 3 days (C) 1 mg fullerene-exposed lung at 3 days (D) 0.2 mg nickel oxide-exposed lung at 3 days. (E) 1 mg fullerene-exposed lung at 1 week, (F) 1 mg fullerene-exposed lung at 1 month. Inflammation including mainly neutrophils was observed in the nickel oxide-exposed group. Mild inflammation was found in lung tissue in the 0.2 mg and 1 mg fullerene-exposed groups.

**Figure 6:**
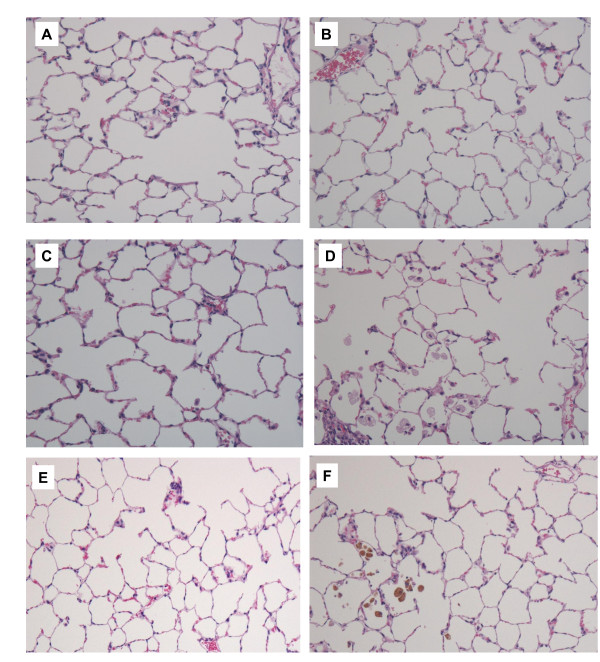
**Hematoxylin and eosin staining of lung sections after intratracheal instillation of fullerenes**. Magnification ×100. (A) Negative control at 3 months (B) 0.2 mg fullerene-exposed lung at 3 months (C) 1 mg fullerene-exposed lung at 3 months (D) 0.2 mg nickel oxide-exposed lung at 3 months. (E) 1 mg fullerene-exposed lung at 6 months, (F) 1 mg fullerene-exposed lung at 6 months. Focal infiltrations of alveolar macrophages with strong pigment deposition were observed in alveoli, although almost neutrophil infiltration disappeared.

Cell infiltration disappeared during the subsequent observation period. In the 1 mg fullerene group, there was significant intra-alveolar infiltration of neutrophils at 3 days and 1 week and there was no difference between the 1 mg fullerene group and the negative control group thereafter. Although not many, focal infiltrations of alveolar macrophages with strong pigment deposition were observed in alveoli after 3 months and 6 months (Figure 6F). There was an inflammatory cell infiltration (mainly neutrophils) in the alveoli and interstitial tissue in the nickel oxide group; this infiltration lasted during the observation period. There were no granuloma, vasculitis, emphysematous change or fibrosis in the fullerene or nickel oxide groups.

#### Morphological features of neutrophils and alveolar macrophages by TEM (Figure 7)

**Figure 7:**
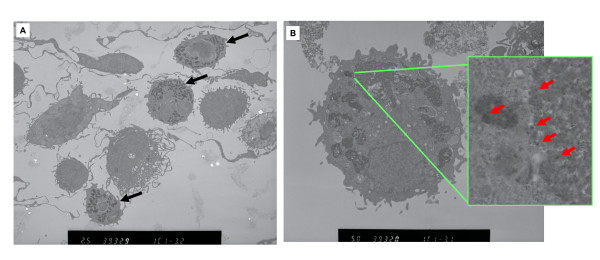
**Alveolar macrophages and neutrophils in 1 mg fullerene-exposed lung using TEM at 3 days after intratracheal instillation**. (A) Neutrophils (B) Alveolar macrophages. Red arrow: Fullerenes. Black colored fine particles (many dispersed black dots) in lysosomes were identified as fullerene particles.

Fine particles were observed more frequently inside alveolar macrophage phagolysosomes in the 1 mg fullerene group, in particular. These black particles and raw fullerene materials exhibited the same diffraction pattern, and fine black particles were identified to be fullerene particles [5]. However fullerene particles were not found in the nucleus and organelles in all test groups including the 1 mg fullerene group. There were neutrophils with granules in the 1 mg fullerene group at 3 days and 1 week.

### 3) Inhalation study

#### Cell count and ALP in BALF (Figure 8)

**Figure 8:**
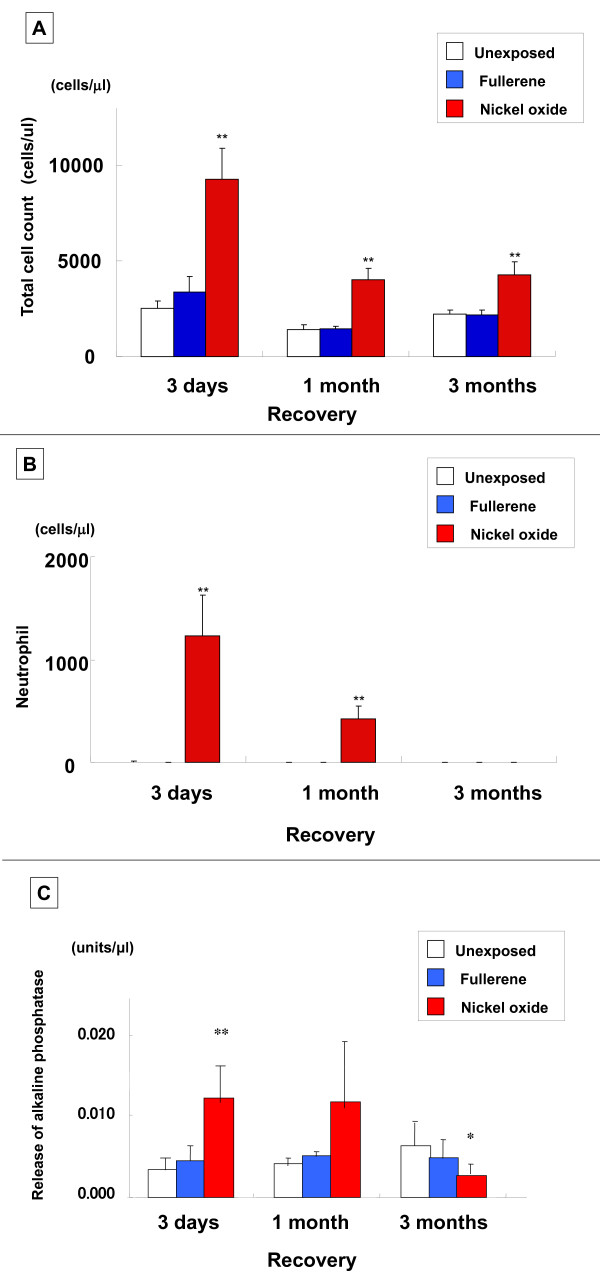
**Analysis of BALF in inhalation study**. (A) Total cell count. (B) Neutrophil cell count. (C) Alkaline phosphatase. Each column and bar represents the mean ± standard deviation of five rats. An asterisk indicates a statistically significant difference of P < 0.05 compared to each unexposed group; double asterisk, of P < 0.01 compared to each unexposed group. Inhalation of fullerenes did not induce an inflammatory response in the rat lungs.

The total cell count of the fullerene inhalation group was almost the same as that of the unexposed group. Also, almost no neutrophil count increase was observed in the unexposed and fullerene groups. The ALP release in BALF was significantly higher in the nickel oxide group, while there was no difference between the fullerene and unexposed groups.

#### CINC concentration in the lung (Figure 9)

**Figure 9:**
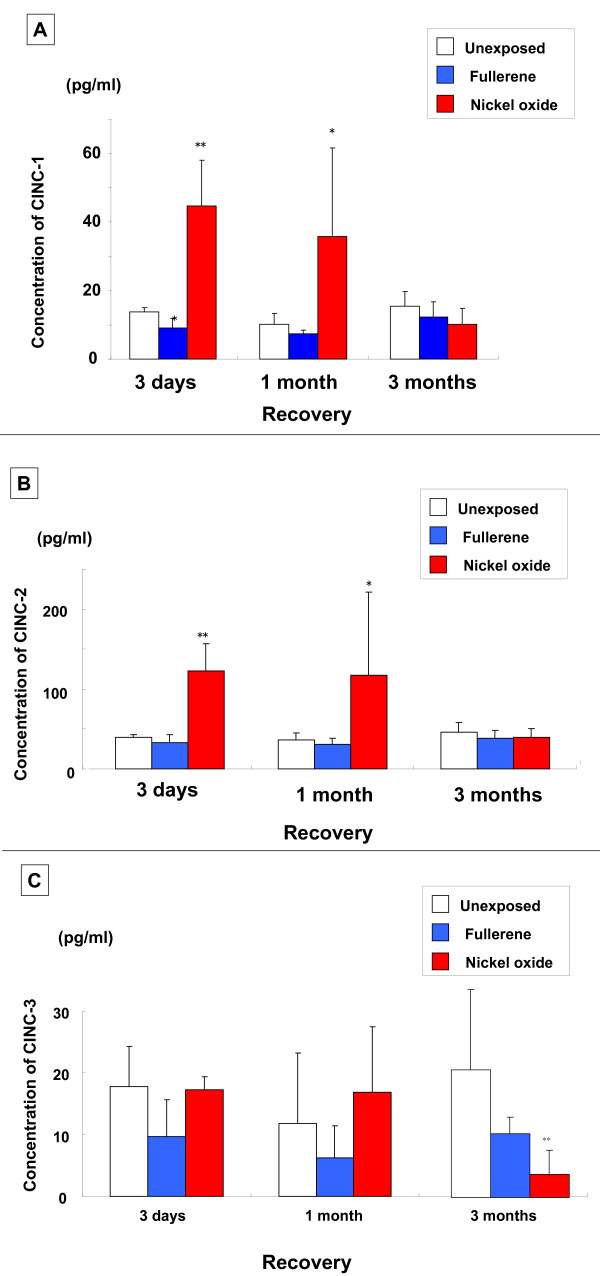
**Concentration of CINC in rat lungs in the inhalation study**. (A) CINC-1. (B) CINC-2αβ. (C) CINC-3. Each column and bar represents the mean ± standard deviation of five rats. An asterisk indicates a statistically significant difference of P < 0.05 compared to each unexposed group; double asterisk, of P < 0.01 compared to each unexposed group. Inhalation of fullerenes did not induce concentration of CINC-1, CINC-2αβ, and CINC-3.

CINC-1 concentration in lung tissue was increased at 3 days and 1 month in the nickel oxide inhalation group as compared to the unexposed group. On the other hand, there was no difference in the CINC-1 concentration between the fullerene inhalation group and the unexposed group during the observation period. As in the case of CINC-1 concentration, CINC-2αβ concentration in lung tissue was not significantly increased in the fullerene inhalation group. No significant difference in CINC-3 concentration in lung tissue was observed among the 3 groups throughout the observation period.

#### Gene expression of CINC mRNA in the lung (Figure 10)

**Figure 10:**
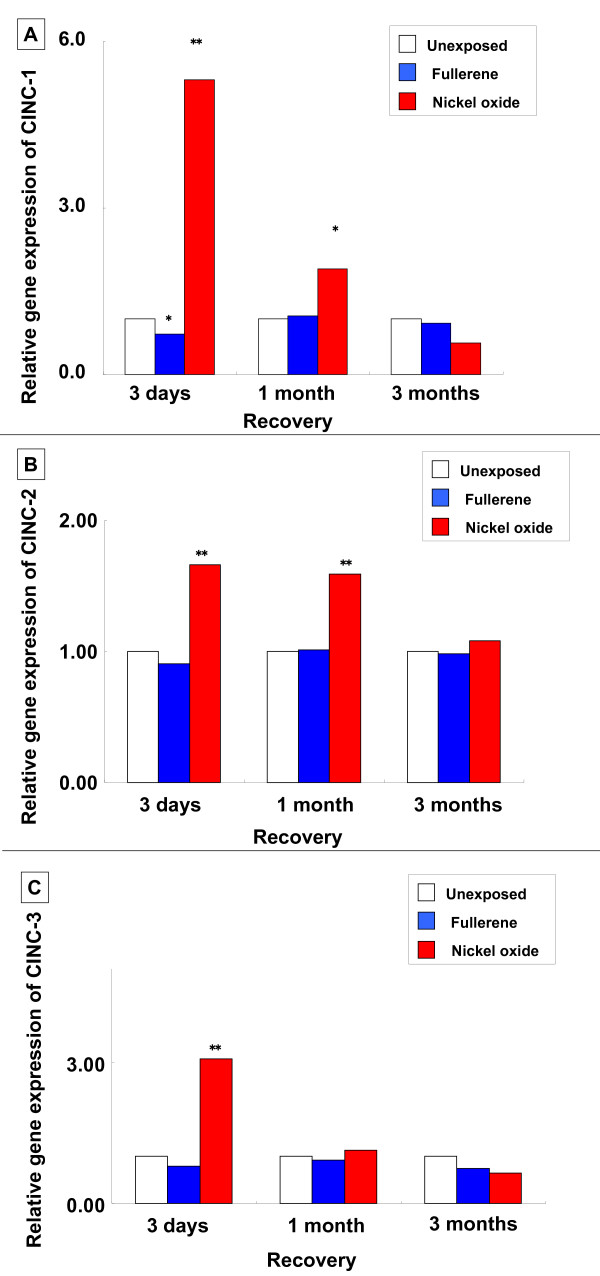
**Gene expression of CINC mRNA in rat lungs in the inhalation study**. (A) CINC-1. (B) CINC-2αβ. (C) CINC-3. Relative gene expression means 2^-ΔΔCT^. (ΔΔC_T_: the difference of the average ΔC_T _value of fullerene-exposed group and the average ΔC_T _of unexposed group). An asterisk indicates a statistically significant difference of P < 0.05 compared to each unexposed group; double asterisk, of P < 0.01 compared to each unexposed group. Inhaled fullerenes did not induce gene expression of CINC-1, CINC-2αβ and CINC-3 mRNA.

There was no significant change of gene expression of CINC-1 between the fullerene and the unexposed groups. CINC-2αβ gene expression was unchanged at a certain significant level in the 0.2 mg nickel oxide instillation, the 0.2 mg fullerene instillation, and the 1 mg fullerene instillation groups. No significant gene expression of CINC-3 was observed between the fullerene and the unexposed groups.

#### Histopathological changes in the lungs (Figure 11)

**Figure 11:**
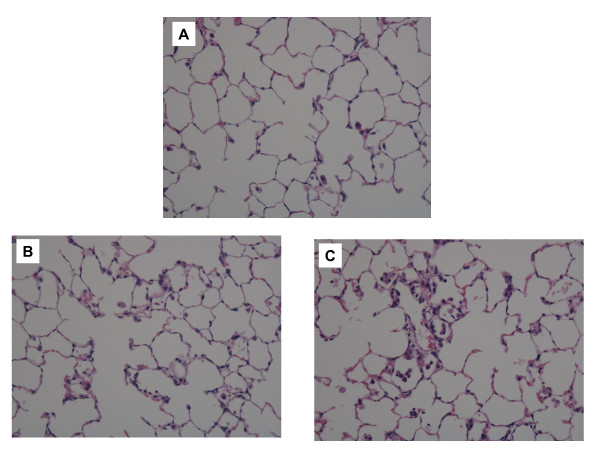
**Hematoxylin and eosin staining of lung sections exposed to fullerenes at day 3 after inhalation**. Magnification ×100. (A) Unexposed lung (B) fullerene-exposed lung (C) nickel oxide-exposed lung. Minimum transient inflammatory response was observed in fullerene-exposed lungs.

Alveolar macrophages with pigment-like material deposition were sometimes observed in alveoli in the fullerene inhalation group, and minimum transient infiltration of alveolar macrophages only was observed in alveoli at 3 days after exposure. On the other hand, in the nickel oxide group, mild infiltration of inflammatory cells, mainly neutrophils and alveolar macrophages, was observed in alveoli and interstitial tissue at 3 days and 1 month after exposure. No granuloma, emphysematous change, or fibrosis was observed during the observation period.

#### Morphological features of neutrophils and alveolar macrophages by TEM (Figure 12)

**Figure 12:**
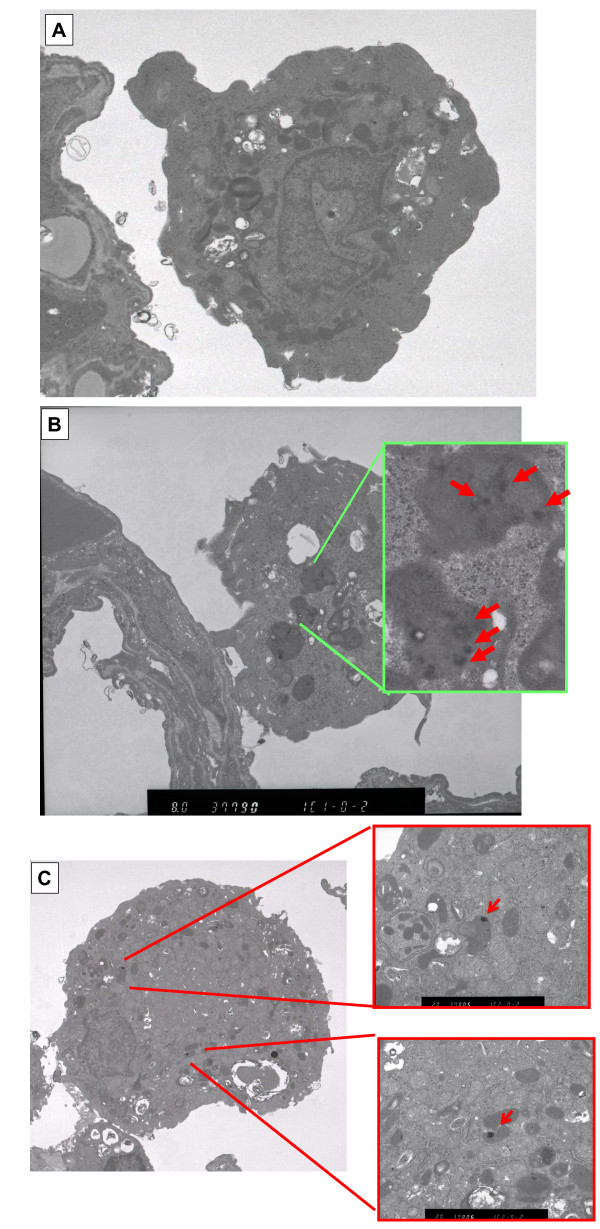
**Alveolar macrophages in fullerene-exposed lungs using TEM at day 3 after inhalation**. (A) Unexposed lung (B) fullerene-exposed lung (C) nickel oxide-exposed lung. Red arrow: Fullerenes. Black colored fine particles (many dispersed black dots) in lysosomes identified as fullerene particles.

Fine black particles were observed inside the phagolysosomes of alveolar macrophages at 3 days in the fullerene-exposed group (Figure 12-B). These black particles were also identified to be fullerenes because they had the same diffraction pattern as the raw materials [5]. There were no fullerene particles in the nucleus and organelles. On the other hand, no fine particles were found in the unexposed group (Figure 12-A).

## Discussion

The same source fullerenes was used for the present intratracheal instillation study and inhalation study; the particle diameter of fullerenes was 33 nm in the intratracheal instillation study and 59 nm in the exposure chamber of the inhalation study. Thus both sizes fell within the range of nanoparticles, 1 nm-100 nm. In addition, because studies have reported that the ratio of fullerene oxide was higher when fullerenes were produced in normal air and this altered its cytotoxicity, we purged the air with nitrogen and confirmed that fullerene oxide was less than 1% [5]. The crystal structure of fullerenes was maintained in the intratracheal instillation and inhalation exposure studies; fullerenes were not denatured by grinding and mixing [5]. The fullerenes we used for the present study were thus evidently pure, nano-size fullerenes.

The intratracheal instillation study of fullerenes showed no significant or transient neutrophil infiltration in BALF and lung tissue at both high and low doses. In our previous intratracheal instillation study of several types of respirable particles, observations were also conducted from day 3 to 6 months after instillation and we found that highly toxic silica and crocidolite showed persistent inflammation, while low toxic titanium dioxide (micron-sized) showed only transient inflammation at the acute phase [17]. Creutzenberg et al. [18] also reported that observations over a 90-day period in their intratracheal instillation study showed a time-dependent increase of inflammation and fibrosis by quartz compounds. The intratracheal instillation study of titanium dioxide by Kobayashi et al. [19] reported an inflammatory cell infiltration up to 1 month after the instillation of less-toxic fine particles and the inflammation disappeared thereafter. Less-toxic particles and the medium alone (negative control) induced a transient neutrophil inflammation at only the acute phase [19,20] while there are materials that caused progressive inflammation and fibrosis such as silica [17,18]. Thus an observation period of 6 months (more than 3 months) seems to be useful for the evaluation of hazardous effects. No persistent inflammation by fullerenes was observed during this period; therefore, the toxicity of fullerenes may be low. Sayes et al. [21] reported that they intratracheally instilled fullerenes with a diameter of 160 nm in suspension and did not find significant toxicity of fullerenes and its derivative, C60 (OH)_24_. This was the first report on a toxicity evaluation of intratracheally instilled fullerenes with a diameter of 100 nm or less. Furthermore, electric microscopic observation in the present study revealed that many fullerene particles were phagocytosed by macrophages but that fullerenes were not transferred to organelles or nuclei, suggesting that there was no damage by direct adhesion of nanoparticle to organelles.

Fullerenes showed an increase of CINC-1 and -2 expressions in the lung tissue, which was transient just as in the case of neutrophil infiltration. The lower dose of nickel oxide in the present study showed a persistent increase in neutrophils along with a persistent increase in CINC-1 and -2 expressions. Several studies reported CINC expression in lung injury models. CINC-1 and CINC-2 expression was increased in an acute pulmonary inflammation model using LPS, and CINC-1 expression was increased in a pulmonary inflammation model using hazardous particles such as diesel particles [13,14]. On the other hand, the results of an intratracheal instillation study using titanium dioxide (micron-size), which is less-hazardous to the lung, revealed a mild and transient increase in CINC-1 and CINC-2αβ expression only at an acute phase [15]. These findings suggest an association between pulmonary neutrophil infiltration and CINC expression in the present study. The level of mRNA was increased for CINC-1 and CINC-3 by fullerenes but CINC-3 expression was dissociated from the protein level. Rao et al. [22] observed an mRNA increase without an increase in protein level after exposure of pulmonary macrophages tosilica. Denaturation of protein may be increased.

In the inhalation study, an inflammatory response mainly with neutrophils was observed in the nickel oxide, while neutrophil infiltration was not observed in the fullerenes. Baker et al. [23] conducted a short-term inhalation study of nano-size fullerenes (3 hours/day, 10 days) and reported that pulmonary inflammation and significant inflammatory cytokines and chemokines in BALF were not observed during 1 week of the observation period. Their result is consistent with ours. The observation period in the present inhalation study was 3 months. Some studies of lung injury by chemical substances reported that inflammation and fibrosis progressed not just after exposure but also in the observation period thereafter [2,24,25]. Bennett et al. [26] reported that fibrosis was caused by chrysotile and silica and not by less-toxic titanium dioxide during the 4-week inhalation period followed by 16 weeks of observation. It was reported that pulmonary inflammation was increased with time after exposure in the inhalation of silica and inflammation was observed to peak after 1 to 3 months [25,27]. The pulmonary deposition of fullerenes in the present inhalation study by analysis of high performance liquid chromatography (HPLC) was less than that of the lower-dose of the intratracheal instillation study [28], and nearly no lesions were observed in the instillation study; these findings are consistent with the results of the inhalation exposure study in which no lesions were observed. CINC-1 and -2 expressions were increased in the nickel oxide and not in the fullerene groups. In the experiment [29], we already analyzed comprehensive gene expression by microarrays and found that CINC expression was increased by the exposure to nickel oxide, while nearly no increase of other neutrophil chemokines was observed. As with the intratracheal instillation study, these results suggest that neutrophil infiltration and CINC-1,-2 expressions are associated with each other in the inhalation exposure study.

There was no foreign body granuloma in the histopathological findings of the inhalation exposure and intratracheal instillation studies of nickel oxide and fullerenes. Foreign body granuloma was often reported, in particular, just after intratracheal instillation. An inhalation exposure study of dispersed single wall carbon nanotubes (SWCNT) reported that granuloma was not found in the lung and fibrosis was caused [2], while granuloma was found in the lung tissue in the case of undispersed SWCNT. It is suggested that the dispersibility of nanoparticles is involved in the initial granuloma formation after instillation or inhalation. The TEM observation showed that phagocytosed fullerenes became finely dispersed granules in alveolar macrophages not only in the inhalation exposure study but also in the intratracheal instillation study.

We measured the release of ALP that is specifically contained in alveolar epithelial cells in both intratracheal instillation and inhalation exposure studies in order to examine the involvement of alveolar epithelial cell injury in lung injury by nano particles. A significant amount of ALP release into BALF was found in the nickel oxide that showed persistent inflammation, while no release was found in the fullerene nanoparticles that showed nearly no inflammation. Sayes et al. [21] reported that there was no significant release of ALP into BALF in the intratracheal instillation of fullerenes and transient release in crystalline silica; their results are similar to our findings. The amount of ALP release was also not persistent in our present study of nickel oxide. Hence initial injury ofthe alveolar epithelium cells may be a factor contributing to subsequent inflammation and fibrosis. An animal model of apoptosis-inducing alveolar epithelium cells reported that severe pulmonary fibrosis was caused by intratracheal instillation of FAS ligand [30], suggesting that only apoptosis in alveolar epithelium cells results in pulmonary fibrosis. Thus, the absence of fullerene-induced injury in alveolar epithelium cells may be one reason for the absence of fullerene-induced injury to the lung.

## Conclusion

Finally, a comparison between the results of the inhalation and instillation studies revealed that inflammatory cell infiltration in BALF, the CINC expression pattern in the lung tissue, and ALP release are similar, except for the high-dose intratracheal instillation. The pathological condition in which nickel oxide lung injury at the acute phase was followed by persistent inflammation tended to be similar in both studies. Thus the pathological features may be similar. These data from the intratracheal instillation and inhalation studies also suggested that well-dispersed fullerenes do not have a strong potential for neutrophil inflammation.

## Materials and methods

### Fullerenes

Bulk high-purity (>99.5%) C60 fullerenes were purchased from Frontier Carbon Corporation (Japan). The manufacturer's specifications indicated a specific surface area of 0.92 m^2^/g. Bulk fullerene material roughly dispersed in 0.1 mg/ml polyoxyethylen sorbitan monooleate (Tween-80, Wako Pure Chemical Industries, Ltd., Japan) was milled in an agate mortar for 30 minutes under a nitrogen atmosphere. The milled fullerene material was suspended with zirconium particles (50 μm) using a high-performance dispersion machine (UAM-15, Kotobuki Industries Co., LTD., Japan) and centrifuged at 8000 × g for 60 min. The concentration was determined by an HPLC system (#1100, Agilent Technologies, Santa Clara, CA). The fullerenes were individually dispersed in this suspension [28]. The mean diameter based on volume and mass by the dynamic light scattering technique (UPA, NIKKISO Co., LTD., Japan) was 33 nm (Figure. 1). This fullerene suspension was used as the directly injected solution in the intratracheal instillation study and for the generation of fullerene nanoparticles in the inhalation study.

### Nickel Oxide

Nickel oxide (20 nm nominal primary diameter, 99.8% purity) was used as a reference material for nanoparticles [31], and was purchased from Nanostructured & Amorphous Materials Inc. The BET specific surface area of the measured sample was 104.6 m^2^/g and the weighted average surface primary diameter (Sauter diameter) was 8.41 nm. The nickel oxide particles were dispersed without any detergents by ultrasonication for 90 minutes using an ultrasonic homogenizer (Model 450, Branson Ultrasonics Corporation, Danbury, USA). The particles were suspendedby centrifugation at 10,000G for 20 min and the recovered supernatant was filtered through a membrane filter with 1 μm diameter pores. The particle size distribution was determined by the dynamic light scattering technique, and the mean diameter based on volume and mass was 26 nm (Figure 1).

### Intratracheal instillation of fullerenes

In the instillation liquid (distilled water containing 0.1% Tween 80), the diameter of fullerene nanoparticles was confirmed as 33 nm (Figure 1). 0.1 mg (0.33 mg/kg) or 0.2 mg (0.66 mg/kg) or 1 mg (3.3 mg/kg) of fullerenes was suspended in 0.4 ml of distilled water including 0.1% Tween 80. 0.2 mg (0.66 mg/kg) and nickel oxide nanoparticles (average agglomerate diameter in the suspension: 26 nm) were suspended in 0.4 ml distilled water. Each material suspension was intratracheally instilled once in Wistar male rats (9 weeks old). The negative control group was exposed to distilled water including 0.1% Tween 80. The animals were dissected at 3 days, 1 week, 1 month, 3 months, and 6 months after instillation.

### Inhalation study of fullerenes

The whole-body exposure system, used to expose rats to fullerenes or nickel oxide nanoparticles, consisted of a pressurized nebulizer and a mist dryer, connected to an exposure chamber (volume: 0.52 m^3^). Same fullerenes and nickel oxide suspensions in the intratracheal instillation study were used for the inhalation study. They were put into the nebulizer and used for the generation of the aerosols [32]. The size and number concentrations of aerosol particles at the exit of the nebulizer and inside the exposure chamber were analyzed in-line using a particle spectrometer consisting of a differential mobility analyzer (DMA) and a condensation particle counter (CPC) (Model 1000XP WPS, MSP Corp., Shoreview, MN) throughout the exposure period. Rats were exposed to the aerosol continuously for 4 weeks (6 hours/days, 5 days/week), and the size distribution and number concentration in the chamber were measured throughout the exposure period. The fullerene aerosol in the chamber had an average mass concentration of 0.12 × 0.03 mg/m^3 ^(0.5 × 0.1 mg/m^3^: including Tween 80) maintained by the isokinetic suction of air through a glass fiber filter, a particle concentration of 4.1 × 10^4 ^particles/cm^3^, and an average geometric diameter of 96 ± 5 nm by DMA and CPC. The nickel oxide nanoparticle aerosol in the chamber had an average mass concentration of 0.2 ± 0.1 mg/m^3^, a particle concentration of 9.2 × 10^4 ^particles/cm^3^, and an average geometric diameter of 59 ± 3 nm. We found through high-resolution transmission electron microscopy that the structure of the fullerene crystals in the aerosol particles remained identical with that in the fullerene suspension. We also confirmed that particle size of fullerenes used in intratracheal instillation and inhalation studies was less than 100 nm by TEM [5,32].

Nine-week-old male Wistar rats were divided into 3 groups: fullerene, nickel oxide nanoparticle, and control groups. The rats inhaled the aerosol for 6 hours a day, 5 days a week, for 4 weeks in a whole-body exposure chamber. The control rats were exposed to only clean air in a same-sized chamber located in the same air-conditioned room. After an exposure period of 4 weeks, the rats were dissected at 3 days, 1 month, and 3 months of recovery.

### Animals after inhalation and intratracheal instillation studies

Each group of 10 animals was divided into 2 subgroups of 5 animals for lung tissue analysis. The first subgroup (5 rats) provided bronchoalveolar lavage, which was collected using physiological saline that was poured through a cannula inserted in the respiratory tract into the right lung, while the left lung was clamped. Three - 10 ml of physiological saline was infused per time and lavage fluid was collected up to 50 ml in total. The left lung was inflated and fixed by intratracheal instillation of 4% paraformaldehyde at 25 cm H_2_O pressure.

The lungs of the second subgroup (5 rats) were homogenized to extract protein and mRNA.

All procedures and animal handling were done according to the guidelines described in the Japanese Guide for the Care and Use of Laboratory Animals as approved by the Animal Care and Use Committee, University of Occupational and Environmental Health, Japan.

### Chemokine measurement of lung tissue and BALF

Lung tissue was homogenized with a T-PER tissue protein extraction reagent, and then centrifuged (1500 × g for 10 min). The protein concentration of the supernatant was measured by the BCA Protein Assay Kit (PIERCE) using Bovine serum albumin. Total protein concentration was adjusted with a final concentration of 500 μg/ml for CINC-1 and CINC-2αβ and 4000 μg/ml for CINC-3. Chemokine concentration was determined by Quantikine Rat CINC-1, CINC-2αβ, and CINC-3 (R&D Systems) (Cat. #RCN100, #RCN200, and #RCN300, respectively) and absorbance at 450 nm was measured by a microplate reader. CINC-1, CINC-2αβ, and CINC-3 in the lung tissue were determined. Alkaline phosphatase (ALP) released in the BALF supernatant was determined by LabAssayTM ALP (Wako Pure Chemical Industries, Ltd. Japan).

### Semiquantitative real-time PCR

RNA was extracted from the lung using RNeasy(R) Mini Kit (50) (QIAGEN Hilden, Germany). Single-strand cDNA was synthesized using High Capacity cDNA Reverse Transcription Kit (Applied Biosystems. CA.).

Real-time PCR and subsequent calculations were performed with the 7500 Real-Time PCR System (Applied Biosystems. Forster. CA. USA), which detects the signals emitted from fluorogenic probes during PCR. Primers and probes were designed according to guidelines from Applied Biosystems with the Primer Express 3.0 software (Applied Biosystems. Forster. CA.). The primer sets were as follows: rat CINC-1; Assay ID Rn00578225_m1, rat CINC-2αβ; Assay ID Rn00593435_m1, rat CINC-3 (MIP-2); Assay ID Rn00586403_m1, and for rat β-actin (RatACTB) Accession number: NM_031144.

Real-time PCR was performed with TaqMan Universal PCR Master Mix reagents. The PCR mixture contained 25 μl of TaqMan Universal PCR Master Mix (2×), 2.5 μl of TaqMan Gene Expression Assays and 17.5 μl of d-water in a total volume of 45 μl. PCR was performed using 5 μl of the first strand cDNA mix. After 2 min at 50°C, to permit UNG cleavage, AmpliTaq Gold was activated by a 10 min incubation at 95°C. Each of the 60 PCR cycles consisted of a 15 s denaturation step at 95°C and a hybridization step, with probes and primers and for DNA synthesis, for 1 min at 60°C.

The average cycle threshold (C_T_) was determined for each group of animals at each time point. Relative gene expression was calculated using the comparative C_T _(ΔΔC_T_: the difference of the average ΔC_T _value of the exposed group and the average ΔC_T _of the control group) method which assesses the difference in gene expression (ΔC_T_: difference between the threshold cycle) between CINC-1, CINC-2αβ and CINC-3 as the target gene and β-actin as the internal standard gene for each sample to generate the ΔΔC_T_. Relative gene expression in the Y axis was then determined by the formula 2^-ΔΔCT^. The relative expression of the average in each group was calculated with respect to the control group in each time point, and the relative expression of the control group was set at 1. Statistical analyses involved comparison of the cycle difference of CINC and β-actin between the exposed and the control groups, and were performed at the ΔC_T _stage.

### Tissue preparation for HE stain

The lungs, which were inflated and fixed by 4% paraformaldehyde, and trachea were resected from the surrounding tissue. The lung tissue was embedded in paraffin, and 5 μm-thick sections were cut from the lobe. The samples were then sectioned and stained with hematoxylin and eosin.

### Processing of lung tissue for transmission electronmicroscope (TEM)

The lung tissues were fixed using glutaraldehyde and osmium tetroxide solution, and then dehydrated in ethanol, and embedded in epoxy resin. The specimens were stained with a 2% uranyl acetate solution and 0.5% lead citrate solution at room temperature. Conventional TEM observation was performed within an H-7000 (Hitachi, Japan) at the acceleration voltage of 80 kV. Energy-filtering TEM observation was performed with an EM922 (Carl Zeiss SMT, Germany), which was equipped with an OMEGA energy filter. Zero-loss filtering, which can increase the scattering and phase contrast of the TEM image was carried out.

### Statistical analysis

Statistical analysis was carried out using the Mann-Whitney test with differences of p < 0.05 considered to be statistically significant.

## Competing interests

The authors declare that they have no competing interests.

## Authors' contributions

Authors YM, IT and JN are responsible for the study design and writing of the manuscript. Authors YM, IT, KF and NS are responsible for data and analysis. Authors MH, AO, TO, TM, KN, CK, MT, MY, MM, MS, WW, KY, SE and KU performed the experiments. All the authors read and approved the final manuscript.
